# Meta-QTL analysis and identification of candidate genes for quality, abiotic and biotic stress in durum wheat

**DOI:** 10.1038/s41598-021-91446-2

**Published:** 2021-06-04

**Authors:** Jose Miguel Soriano, Pasqualina Colasuonno, Ilaria Marcotuli, Agata Gadaleta

**Affiliations:** 1 Sustainable Field Crops Programme, IRTA (Institute for Food and Agricultural Research and Technology), 25198 Lleida, Spain; 2 Department of Agricultural and Environmental Science, University of Bari ‘Aldo Moro’, Via G. Amendola 165/A, 70126 Bari, Italy

**Keywords:** Quantitative trait, Plant breeding

## Abstract

The genetic improvement of durum wheat and enhancement of plant performance often depend on the identification of stable quantitative trait loci (QTL) and closely linked molecular markers. This is essential for better understanding the genetic basis of important agronomic traits and identifying an effective method for improving selection efficiency in breeding programmes. Meta-QTL analysis is a useful approach for dissecting the genetic basis of complex traits, providing broader allelic coverage and higher mapping resolution for the identification of putative molecular markers to be used in marker-assisted selection. In the present study, extensive QTL meta-analysis was conducted on 45 traits of durum wheat, including quality and biotic and abiotic stress-related traits. A total of 368 QTL distributed on all 14 chromosomes of genomes A and B were projected: 171 corresponded to quality-related traits, 127 to abiotic stress and 71 to biotic stress, of which 318 were grouped in 85 meta-QTL (MQTL), 24 remained as single QTL and 26 were not assigned to any MQTL. The number of MQTL per chromosome ranged from 4 in chromosomes 1A and 6A to 9 in chromosome 7B; chromosomes 3A and 7A showed the highest number of individual QTL (4), and chromosome 7B the highest number of undefined QTL (4). The recently published genome sequence of durum wheat was used to search for candidate genes within the MQTL peaks. This work will facilitate cloning and pyramiding of QTL to develop new cultivars with specific quantitative traits and speed up breeding programs.

## Introduction

Durum wheat is an important cereal crop grown in a wide range of agricultural regions. The Mediterranean basin represents more than half of the world’s durum wheat growing area, but it is also grown in the northern plains of the United States and Canada, the desert areas in the southeast United States and northern Mexico, and to a minor extent in other regions.

(International Grain Council, https://www.igc.int/en/default.aspx), all of which are characterized by low rainfall. Wheat is successful due to its wide adaptation to local environments and good processing propertiesin the Mediterranean, soil water availability is a limiting factor incereal crop productivity,and biotic and abiotic stress may strongly affect wheat quality)^1^.

Water scarcity, often associated with high temperatures during the grain filling period, severely affects durum wheat quality and yield^2,3^. At this stage of the crop cycle,lack of water and high temperaturesreduce photosynthesis and the source-to-sink transportation of photosynthates in the caryopsis, thereby affecting the formation of the seed proteome. In contrast, excess moisture improves the yield by increasing starch concentrations in the caryopsis and therefore reducing the protein content. A crucial factor in determining the quality of semolina is the seed protein content (and its composition)^4–6^. In addition, as reported in^7^, genotype × environment effects can also alter the composition of the reserve proteome.

Therefore, improving breeding programs aim tocombine the highest number of desirable traits in the same genotype. Combining all the most favourable alleles in one cultivar translates into advantages for the miller and the consumer. High-quality kernels produce good quality flour with a balanced protein profile that guarantees high quality doughs and therefore end products with adequate texture and structure that meet consumer requirements. Certain traits not only satisfy consumers but also have nutritional value. An example is the colour of semolina: consumers generally appreciatea yellow pigmentation, which alsoindicates a high level of carotenoid pigments in the kernel. Combining the highest number of genes involved in carotenoid trait expressionis thereforea tool for both improving the nutritional value of wheat and satisfying consumers^8^.

In 2019 nearly 16 million tons of pasta were produced worldwide. Italy is the greatest consumer, with near 24 kg of pasta consumed per person each year (https://internationalpasta.org/). There is increasing awareness of the importance of wheat-based products in a healthy diet, and producers are identifying and exploiting natural variations in bioactive compounds. However, in some cases natural variations in a trait may be limited in extent or be difficult to exploit, so that other approaches may be required, as in this case. The most important targets of this type of approach are currently minerals, resistant starch, antioxidant compounds, carotenoids, protein content and dietary fibre. As mentioned earlier, quality is directly linked to biotic and abiotic stress. In recent years many quantitative trait loci (QTL) studies have focused on these traits, such as fiber content QTL in Marcotuli et al.^9^, root and shoot morphological traits in Iannucci et al.^10^, and many others reviewed in Colasuonno et al.^11^. These studies identified hundreds of QTL in different mapping populations with different types of markers besides. To identify the genome regions most involved in trait variationand the major, stable QTLs affecting these traits, the QTL meta-analysis approach developed by Goffinet and Gerber^12^ can help narrow down QTL regions, identify candidate genes and tackle map-based cloning strategies.

This approach allows the integration of independent QTL studies in a consensus mapor reference genome of the species. QTL meta-analysis is a powerful tool for discovering genome regions most frequently implicated in trait variation and forreducing the QTL confidence intervals, thereby enhancing the detection of candidate genes for positional cloning^13^. To identify meta-QTL (MQTL) for their use in marker-assisted breeding, Loffler et al.^14^ defined three criteria: (1) the MQTL must have a small supporting interval, (2) include a high number of original QTL, and (3) those QTL must have a large effect on the phenotypic variance explained.

Many of the traits mentioned above and analysed in the present paper are polygenic traits, and associated QTL have been located on all the tetraploid wheat chromosomes.

Meta-QTL (MQTL) analysis is a good instrument for studying many traits at once and finding the consensus, robust QTL region through the use of data reported in multiple studies for the reliability of their location and effect across different genetic backgrounds and environments, as well as to refine QTL positions on a consensus map^12^.The recent sequencing of the ‘Svevo’ durum wheat genome has enabled the identification of consensus genomic regions, the study of relationships among candidate genes within QTL, and the identification of pleiotropic effects among them^15^.

There are many examples in which MQTL analysis has also been successfully used to detect consensus QTL regions in wheat: root-related traits^13,16^, pre-harvest sprouting tolerance^17^, ear emergence^18,19^, resistance against Fusarium head blight^20–22^, plant height^23^, grain dietary fiber content^24^, seed size and shape^25^, yield-contributing traits^24,26–28^, resistance to leaf rust^29^; pasta-making quality^30^; potassium use efficiency^31^; drought tolerance^32^; tan spot resistance^33^. The objective of the present study was to focus on MQTL analysis of durum wheat progenies using a highly saturated consensus map from Macaferri et al.^15^, taking into account a high number of traits in order to identify major regions and possible pleiotropic gene effects.

## Results

### QTL distribution and projection

A total of 41QTL studies for quality, abiotic and biotic stress reported inColasuonno et al.^11^ were analysed, including 36 different traits (Table 1). The studies involved 34 different mapping populations, including 53 different parental accessions (Table 2). QTL projection was carried out using only QTL having the same flanking markers in the consensus map. A total of 368 QTL distributed on all 14 chromosomes (genomes A and B) were projected: 171 corresponded to quality-related traits; 127 to abiotic stress, and 71 to biotic stress.

**Table 1:** Traits for biotic stress, abiotic stress and quality reported in the QTL meta-analysis.

Trait	Description
**Biotic stress**	
CP	Clavicepspurpurea resistance
FHB	Fusarium head blight resistance
LR	Leaf rust resistance
LS	Loose smut resistance
PM	Powdery mildew resistance
SBCMV	Soil-borne cereal mosaic virus resistance
SR	Stem rust resistance
STB	*Septoriatritici* blotch resistance
YR	Yellow rust resistance
**Abiotic stress**	
CC	Chlorophyll content
CIR	Carbon isotope ratio
CL	Coleoptile length
DB	Dry biomass
FLRI	Flag leaf rolling index
NDVI	NDVI
OP	Osmotic potential
PDL	Length of the ear peduncle
RRT	Root related traits
SPAD	Chlorophyll content
**Quality**	
AX	Arabinoxylan
BG	β-glucan
Fb	Flour yellow colour
GCaC	Grain calcium concentration
GCuC	Grain copper concentration
GFeC	Grain iron concentration
GKC	Grain potassium concentration
GMgC	Grain magnesium concentration
GMnC	Grain mangnese concentration
GPC	Grain protein content
GSC	Grain sulphur concentration
GSeC	Grain selenium concentration
GseY	Grain selenium yield
GZnC	Grain zinc concentration
PGC	Phosphorus grain concentration
SV	SDS-sedimentation volume
YPC	Yellow pigment content

**Table 2:** Mapping populations used in the study and related references, including the years when experiments were done and the number of environments (Env).

References	Cross	Type	Size	Trait	N QTL	Years	Env
^50^	Langdon × G18-16	RIL	156	CIR, OP, CC, FLRI	6, 9, 7, 9	2004	2
^51^	Kofa × Svevo	RIL	247	PDL, SPAD, NDVI	4, 3, 5	2004, 2005	8
^52^	Omrabi5 × Belikh2	RIL	114	CL, RRT	5, 1	2009	2
^53^	Colosseo × Lloyd	RIL	176	RRT	28	–	1
^53^	Meridiano × Caludio	RIL	181	RRT	32	–	1
^10^	Simeto × MolliseColli	RIL	136	RRT	18	–	1
^54^	Strongfield × Blackbird	DH	85	FHB	2	–	1
^55^	LDN × LDN-Dic7A	RIL	118	FHB	1	2004, 2005	3
^56^	Colosseo × Lloyd	RIL	176	LR	1	2006, 2007	1
^57^	Meridiano × Claudio	RIL	181	SBCMV	1	2007, 2008	1
^58^	DS × Td161	BC	134	FHB	1	2006, 2008	2
^58^	Floradur × Td161	BC	129	FHB	3	2006, 2008	2
^58^	Helidur × Td161	BC	126	FHB	1	2006, 2008	2
^59^	Kristal × Sebatel	RIL	85	SR	7	2008–2010	2
^60^	Simeto × Levante	RIL	180	SBCMV	7	2008, 2009	1
^61^	BGRC3487 × 2 * DT735	RIL	160	FHB	2	2008–2010	2
^62^	Cirillo × Neodur	RIL	146	SBCMV	2	2008	1
^63^	Wollaroi × Bansi	RIL	92	YR	2	2007–2009	1
^64^	Gerizim × Helidur	RIL	103	FHB	1	2008, 2009	2
^65^	Langdon × G18-16	RIL	157	PM	4	–	1
^66^	Latino × MG5323	RIL	110	LR	3	–	1
^67^	Ben × PI41025	RIL	200	FHB	3	2010–2012	1
^68^	Sumai-3 × Saragolla	RIL	135	FHB	11	2012, 2013	2
^69^	Karur × DBC-480	RIL	111	FHB	1	2013–2015	1
^70^	Strongfield × Blackbird	DH	90	LS	2	2011, 2012	1
^71^	Kofa × W9262-260D3	DH	155	YR	1	2013	1
^72^	Joppa × 10Ae564	RIL	205	FHB	3	2015, 2016	2
^73^	Rusty × PI 192051-1	RIL	180	LR	5	2017	2
^74^	Ben × Tunisian 108	BIL	171	FHB	3	2010, 2011	2
^75^	Greenshank × AC Avonlea	DH	132	CP	4	–	2
^76^	UC1113 × Kofa	BP	93	YPC	4	2003–2006	2
^50^	Langdon × G18-16	RIL	152	GCaC, GCuC, GFeC, GKC, GMgC, GMnC, GPC, GSC, GZnC, PGC	5, 10, 10, 8, 2, 2, 4, 5, 6, 3	2004	2
^77^	DT695 × Strongfield	DH	185	GPC	6	2002, 2003, 2005	3
^78^	Latino × Primadur	BP	121	YPC	4	2006, 2008	3
^79^	UC1113 × Kofa	RIL	93	GPC, SV	8, 10	2006, 2007	3
^80^	UC1113 × Kofa	BP	93	F, YPC	7, 6	2006, 2007	3
^81^	Svevo × Ciccio	BP	120	YPC	7	2006, 2007	2
^82^	Duilio × Avonlea	RIL	134	BG	2	2014	2
^83^	Langdon × G18-16	RIL	152	GSeC, GSeY	9, 6	2005, 2007	2
^8^	Colosseo × Lloyd	BP	176	YPC	9	–	–
^8^	Kofa × Svevo	BP	249	YPC	9	–	–
^8^	Meridiano × Claudio	BP	181	YPC	6	–	–
^84^	Svevo × Y12-3	RIL	208	GPC	12	2014, 2015	3
^4^	Saragolla × 02-5B-318	RIL	135	GPC	4	2015–2017	1
^61^	Pelissier × Strongfield	DH	162	SV	6	2008–2010	2

**Figure 1:**
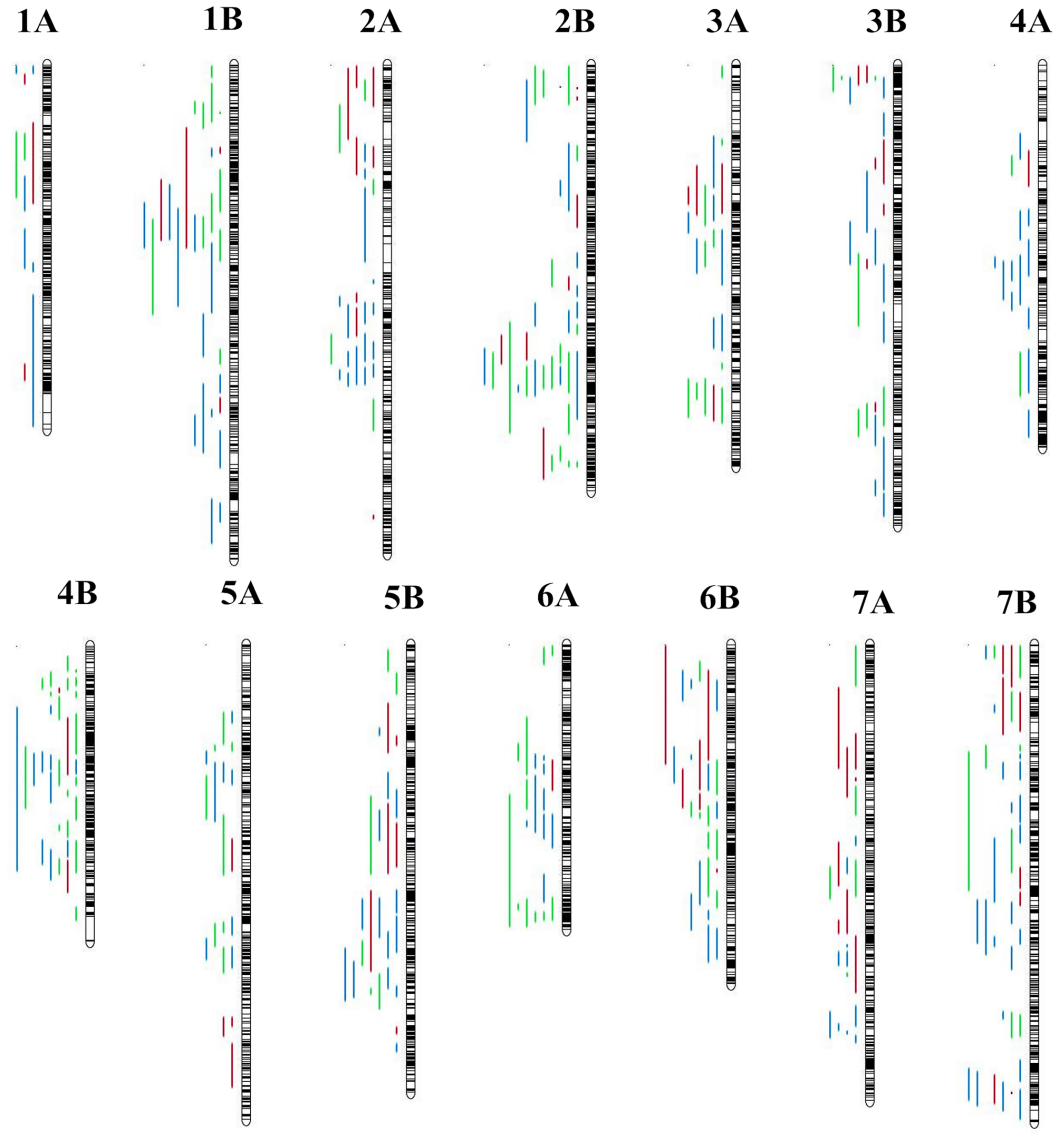
QTL distribution along durum wheat genome chromosomes A and B. Colour code: green: abiotic stress QTL; orange: biotic stress QTL; blue: quality QTL. Black bars within chromosomes represent marker density.

Differences in the number of projected QTL were observed not only among all the seven homoeologous groups, but also among individual chromosomes within a homoeologous group (Fig. 1).The number of projected QTL per genome was 144 (39%) and 244 (61%) for genomes A and B, respectively.The number of QTL per chromosome ranged from 11 in chromosome 1A to 40 in chromosomes 2B and 7B, with an average of 26 QTL per chromosome.

**Figure 2:**
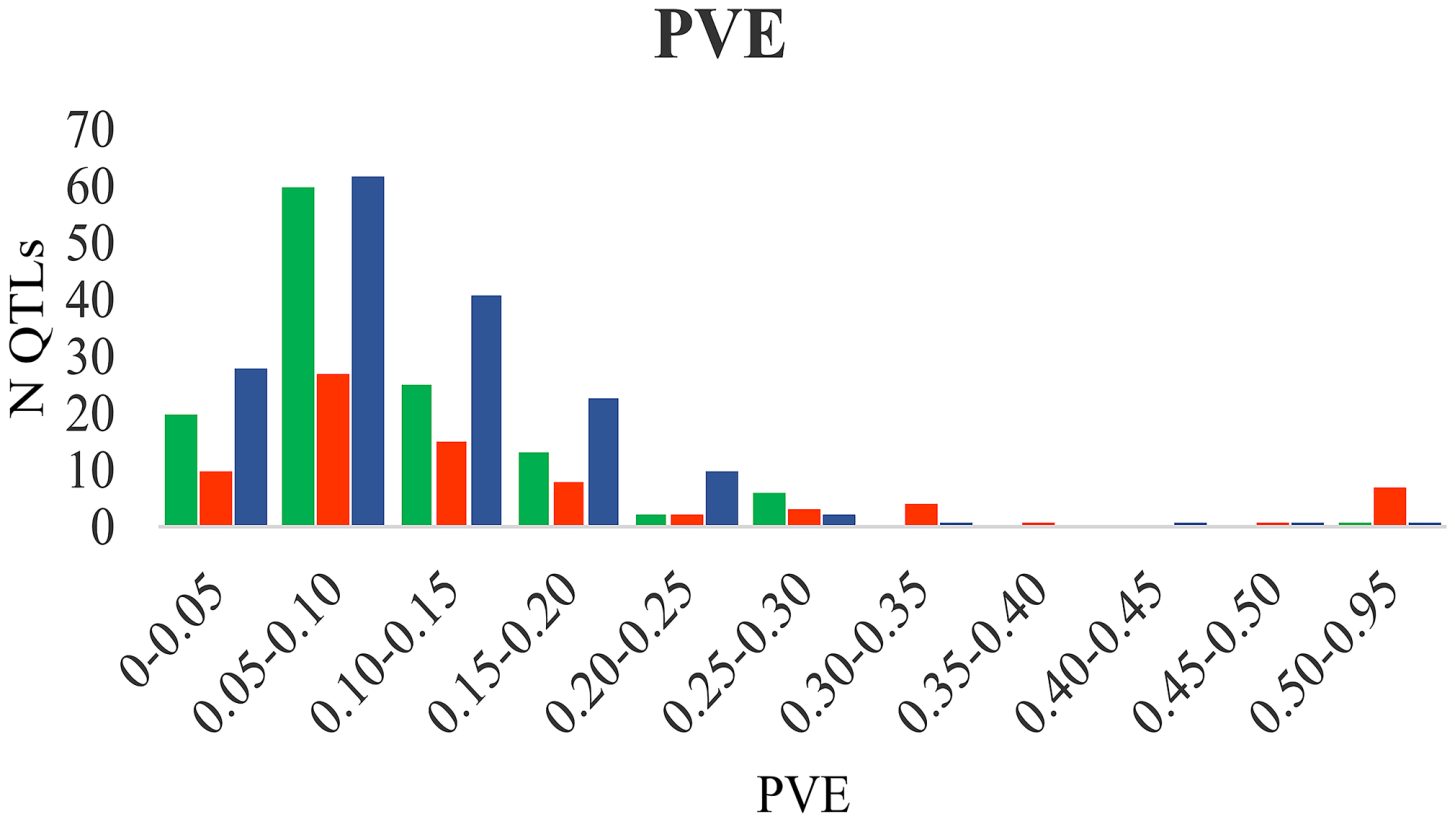
Phenotypic variance explained by original QTL. Colour code: green: abiotic stress QTL; orange: biotic stress QTL; blue: quality QTL.

The means of the proportion of phenotypic variance explained (PVE) by the original QTL showed a similar pattern among the traits, with 63%, 53% and 48% of the QTL showing a PVE < 0.10, for abiotic stress, biotic stress and quality respectively (Fig. 2).

**Figure 3:**
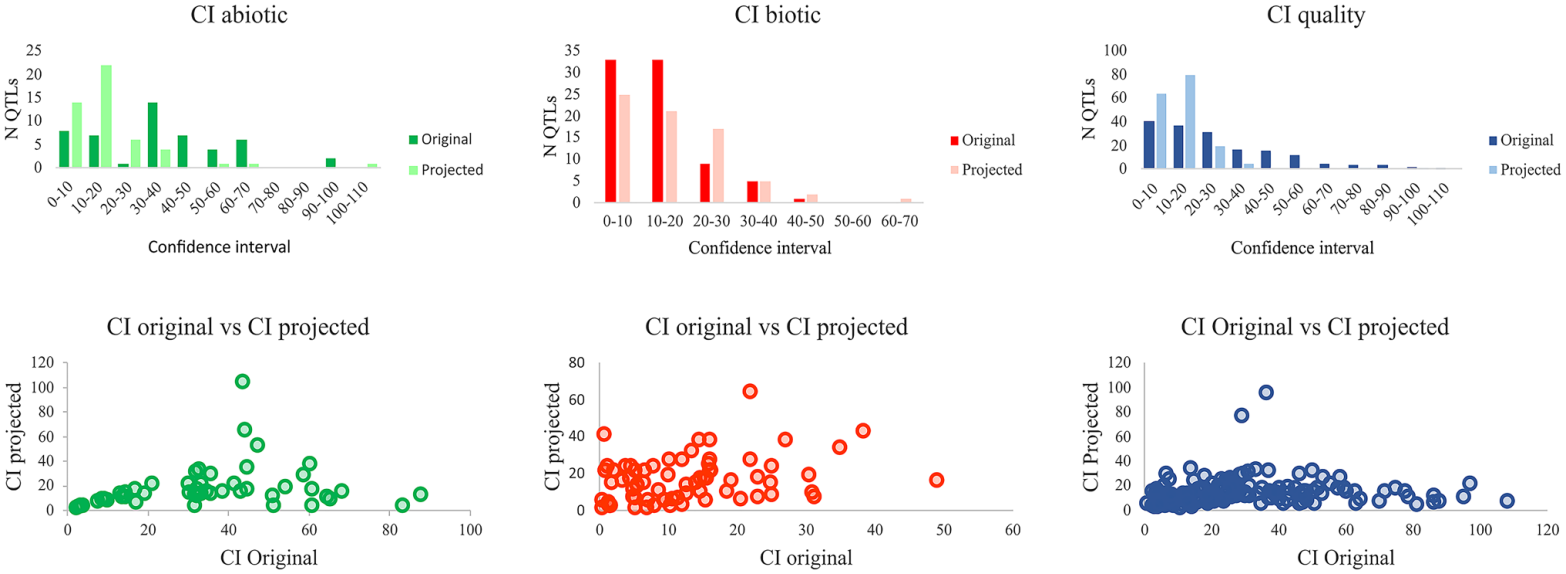
Comparison of confidence intervals for original and projected QTL and their correlation for the different traits.

When the confidence interval (CI) was not reported in the original studies, it was calculated as the distance between the flanking markers. The CIs in the projected QTL were estimated at 95% using the empirical formula proposed by Guo et al. (2006). Comparison between CIs in original and projected QTL (Fig. 3) revealed clear differences for abiotic stress and quality traits. Most of the projected QTL for these traits showed lower CIs, with respective mean values of 35 cM and 18 cM for original and projected abiotic stress CIs and of 28 cM and 14 cMfor original and projected quality traits. In the case of biotic stress traits, instead, the original QTL showed lower CIs (mean 13 cM) than the projected QTL (mean 17 cM). For abiotic stress, 69% of the original QTL had CIs greater than 20 cM, whereas 73% of the projected QTL had CIs lower than 20 cM. For biotic stress traits, 79% and 65% of the original and projected QTL yielded CI values lower than 20 cM, respectively. Lastly, for quality traits, 54% of the original QTL had CIs greater than 20 cM, whereas 85% of the projected QTL yielded CIs lower than 20 cM.

### QTL meta-analysis

Of the 368 QTL projected onto the consensus map of Maccaferri et al. (2015), 318 were grouped in 85 meta-QTL (MQTL) (Table 3) and 24 remained as single QTL not overlapping with MQTL. The remaining 26 QTLwere not assigned to any MQTL either, because their CI overlapped with different MQTL or because the predicted QTL peaks were not included within any MQTL. They were not considered as single QTL, as their CI overlapped with MQTL.

**Table 3:** Characterization of MQTL.

MQTL	Peak	N QTL	Traits	CI left (cM)	CI right (cM)	Left closest marker	Position (bp)	Right closest marker	Position (bp)
durumMQTL1A.1	3.5	3	FHB, YPC, GPC	2.1	5.0	BS00064204_51	3,574,024	wsnp_Ex_c2868_5293485	7,130,925
durumMQTL1A.2	46.1	4	LR, SR, CC, YPC	41.2	50.9	RAC875_c17283_453	48,029,342	Tdurum_contig48416_335	362,986,005
durumMQTL1A.3	92.7	2	AX, SV	90.1	95.4	RAC875_c16149_298	509,524,234	wsnp_Ex_c3258_6004611	521,653,795
durumMQTL1A.4	142.0	2	LR, YPC	139.3	144.6	wsnp_Ex_c3201_5910659	571,925,048	CAP12_rep_c5332_341	576,427,845
durumMQTL1B.1	16.9	4	RRT	16.5	17.4	wPt-5006	16,362,423	wPt-0655	17,484,592
durumMQTL1B.2	29.3	2	LR, YPC	28.2	30.5	TA002065-1430	52,576,257	Kukri_c5861_360	65,241,426
durumMQTL1B.3	55.3	10	FHB, YPC, RRT, GPC, CL, AX, SV	52.9	57.8	BS00069723_51	449,298,414	RAC875_c92464_53	478,463,076
durumMQTL1B.4	95.3	2	RRT, GPC	92.5	98.1	wsnp_Ex_rep_c71376_70138381	585,368,279	BS00064162_51	594,840,328
durumMQTL1B.5	117.6	5	GseC, FHB, YPC, GPC	116.2	119.0	Kukri_rep_c97349_140	626,782,208	BS00089790_51	633,135,607
durumMQTL1B.6	151.3	2	GseY, YPC	149.8	152.7	D_GBUVHFX01AHO3C_336	661,200,398	IAAV6011	664,610,714
durumMQTL2A.1	8.2	4	FHB, SBCMV, BG, RRT	4.9	11.6	RFL_Contig4030_493	4,105,954	D_contig79877_194	10,965,955
durumMQTL2A.2	39	4	LR, BG, FHB, NDVI	36.1	42.3	Kukri_c27040_309	29,530,680	Ku_c23118_149	34,130,605
durumMQTL2A.3	50.84	3	NDVI, GPC, SPAD	48.8	52.9	Tdurum_contig32692_271	38,336,059	Tdurum_contig46797_585	43,936,608
durumMQTL2A.4	104.6	6	YPC, SR, NDVI	103.1	106.1	gwm95	156,761,990	Kukri_c52614_291	192,760,487
durumMQTL2A.5	126.1	4	FLRI, YPC, GZnC	123.6	128.5	Tdurum_contig42540_843	603,597,766	Tdurum_contig101781_53	608,746,813
durumMQTL2A.6	135.2	4	GPC, GZnC, LR, YPC, GFeC	133.5	136.9	BobWhite_c34273_67	644,481,819	wsnp_JD_c514_781859	671,466,946
durumMQTL2B.1	10.8	5	RRT, SBCMV, BG, SPAD, NVDI	10.4	11.2	BS00081871_51	11,131,675	BS00085748_51	9,712,138
durumMQTL2B.2	59.0	2	GseY, GPC	55.4	62.7	Tdurum_contig74936_133	79,053,860	Tdurum_contig59780_988	99,231,827
durumMQTL2B.3	91.0	2	NDVI, BG	87.5	94.6	RAC875_c5080_915	201,176,819	RFL_Contig3353_125	404,189,154
durumMQTL2B.4	102.9	2	LR, GFeC	100.5	105.3	Ra_c106383_270	446,141,376	RAC875_c992_370	493,056,835
durumMQTL2B.5	115.0	3	GFeC, SV, YPC	112.0	118.0	Excalibur_c84741_99	537,614,290	wsnp_Ex_c114_229879	570,335,910
durumMQTL2B.6	126.7	4	RRT, GPC, NDVI, LR	124.4	128.9	GENE-1352_214	603,649,910	IAAV5675	633,838,200
durumMQTL2B.7	147.5	11	GseY, LR, FLRI, RRT, GFeC	145.8	149.1	BobWhite_c27184_148	697,868,929	Tdurum_contig17826_338	714,626,523
durumMQTL2B.8	183.3	5	NDVI, SPAD, PDL, PM	183.2	183.5	BS00065302_51	778,068,615	BS00083998_51	778,539,953
durumMQTL3A.1	61.8	5	FHB, DB, NDVI, SBCMV	58.4	65.1	Tdurum_contig43850_140	117,216,598	BS00063531_51	282,116,044
durumMQTL3A.2	83.7	4	LR, PDL, GFeC, STB	80.4	87.1	Tdurum_contig59531_914	532,975,275	wsnp_Ku_c5378_9559013	560,465,228
durumMQTL3A.3	123.5	2	GSeC, AX	117.7	129.4	Kukri_c25064_120	649,262,262	RAC875_c22641_993	739,903,219
durumMQTL3A.4	150.2	5	RRT	148.9	151.6	Tdurum_contig42495_389	705,563,307	wsnp_Ex_c9377_15572157	707,002,492
durumMQTL3B.1	6.3	7	SPAD, NDVI, RRT, LR, PDL	5.5	7.2	Kukri_rep_c88385_226	6,221,552	wsnp_Ex_c40595_47620787	6,886,922
durumMQTL3B.2	45.7	2	FHB	42.7	48.8	BobWhite_c62702_587	54,867,872	GENE-1900_115	67,253,287
durumMQTL3B.3	67.0	2	SBCMV, YPC	63.9	70.1	RFL_Contig3857_548	133,186,432	RAC875_c79844_323	167,491,237
durumMQTL3B.4	91.7	5	SR, SV	89.2	94.2	BobWhite_rep_c61884_158	516,937,853	Kukri_c15521_2027	553,129,195
durumMQTL3B.5	159.9	5	RRT, YR, CL, RRT	157.7	162.1	BS00063624_51	772,236,110	Kukri_c3243_1016	776,834,461
durumMQTL3B.6	196.6	3	YPC	195.2	198.1	RAC875_c111148_585	817,039,063	wsnp_BE444579B_Ta_2_2	818,195,973
durumMQTL4A.1	44.0	3	GPC, CIR, LR	40.3	47.7	IACX62	46,560,597	wsnp_Ex_c30989_39836034	88,066,155
durumMQTL4A.2	71.6	2	LR, GCuC	67.8	75.4	wsnp_BF484585A_Td_2_1	572,071,933	TA005643-0627	583,594,297
durumMQTL4A.3	92.7	5	GPC, YPC, GCuC	90.2	95.2	Ku_c6779_1381	604,660,434	Tdurum_contig61343_177	608,153,268
durumMQTL4A.4	112.4	2	SR, GCuC	108.7	116.1	wsnp_Ex_c41313_48161689	622,734,003	BobWhite_c10610_149	635,190,258
durumMQTL4A.5	154.5	3	SR, CC	151.2	157.9	Kukri_c13761_379	702,329,076	wPt-9196	707,410,962
durumMQTL4B.1	11.6	2	RRT, CL	10.5	12.6	Tdurum_contig29961_68	11,083,105	wsnp_Ex_c10347_16946522	12,042,854
durumMQTL4B.2	17.1	4	RRT, CL	15.5	18.7	GENE-4933_489	13,403,076	Tdurum_contig68677_480	18,074,456
durumMQTL4B.3	21.7	3	FHB, RRT	20.7	22.7	Kukri_c34633_69	20,795,117	BS00022431_51	23,204,984
durumMQTL4B.4	30.3	2	GPC, NDVI	28.0	32.7	Tdurum_contig75738_113	26,056,520	IACX47	30,112,862
durumMQTL4B.5	54.6	6	GMnC, GPC, AX, SV, GMnC	51.5	57.8	TA006298-0500	383,231,914	wsnp_Ex_c16083_24512551	453,294,222
durumMQTL4B.6	64.4	3	GCuC, RRT, GseY	62.9	65.9	Tdurum_contig24612_209	504,883,147	Kukri_c322_1394	524,075,645
durumMQTL4B.7	82.8	2	RRT	81.0	84.6	wsnp_Ex_c23638_32875196	607,834,125	RAC875_c14455_1148	621,516,171
durumMQTL4B.8	98.14	6	YPC, GCuC, RRT	95.8	100.5	Tdurum_contig8322_966	646,421,893	wsnp_Ex_c14138_22066009	652,716,927
durumMQTL5A.1	48.6	3	YPC, RRT	47.1	50.0	D_GA8KES402GAVSF_317	111,907,960	Kukri_c25407_645	331,277,629
durumMQTL5A.2	61.6	4	GseC, YPC, AX	58.6	64.6	Tdurum_contig5481_369	395,919,866	BS00022110_51	401,330,652
durumMQTL5A.3	96.1	2	SBCMV, SR	89.1	103.1	wsnp_BE443745A_Ta_2_1	439,542,927	BobWhite_rep_c50888_306	468,004,808
durumMQTL5A.4	131.1	3	GCaC, CIR, RRT	128.7	133.5	CAP7_c4800_276	527,044,675	Tdurum_contig60421_74	529,441,492
durumMQTL5A.5	143.2	3	GseY, OP, GPC	139.9	146.6	BobWhite_c40643_370	537,480,079	Excalibur_c26671_57	553,019,889
durumMQTL5A.6	175.6	3	FHB	174.9	176.3	Excalibur_c4083_874	607,909,980	Ku_c24141_700	610,522,247
durumMQTL5B.1	9.9	2	NDVI, SPAD	5.9	13.8	wsnp_Ku_c10586_17464696	9,787,752	Tdurum_contig92396_380	17,218,406
durumMQTL5B.2	42.0	3	GPC, CP, FHB	40.3	43.8	Excalibur_c57167_475	84,059,870	Excalibur_c15262_2304	327,019,780
durumMQTL5B.3	69.3	2	Fb	64.4	74.2	Tdurum_contig28754_218	439,553,088	Kukri_c10296_1512	475,596,832
durumMQTL5B.4	87.1	4	FLRI, PM, GCaC, SR	81.5	92.7	GENE-3437_68	489,178,563	Tdurum_contig53926_455	514,395,841
durumMQTL5B.5	122.2	3	GCaC, GPC, YPC	118.0	126.4	wsnp_Ex_c13485_21225504	559,774,294	wsnp_Ra_c39562_47242455	576,849,094
durumMQTL5B.6	139.0	3	GMgC, CC, GCaC	135.0	143.1	Excalibur_rep_c88310_1394	588,418,255	RFL_Contig3835_475	604,697,415
durumMQTL5B.7	159.3	5	YPC, CIR, OP	157.8	160.8	Tdurum_contig56335_223	643,149,387	IACX3775	649,698,450
durumMQTL6A.1	2.3	2	NDVI	0.0	4.9	BobWhite_c43135_397	1,819,265	Tdurum_contig41990_1324	7,436,293
durumMQTL6A.2	53.7	6	YPC, CL, LR, RRT, SR	52.4	55.0	Excalibur_c33110_52	323,649,274	wsnp_Ex_c35545_43677480	443,168,317
durumMQTL6A.3	81.3	6	YPC, SV, CIR	79.6	83.1	BS00023893_51	552,510,396	BS00065082_51	553,838,425
durumMQTL6A.4	123.6	6	RRT, NDVI, AX	123.4	123.8	BobWhite_c24258_496	602,232,048	RAC875_c27781_591	602,503,159
durumMQTL6B.1	18.1	5	GKC, RRT, YPC, GKC	15.8	20.3	Excalibur_c72517_251	13,113,351	Kukri_rep_c103034_636	17,165,695
durumMQTL6B.2	61.0	5	GKC, OP, FHB, NDVI	56.8	65.3	Tdurum_contig48689_514	126,247,427	BS00073879_51	146,626,621
durumMQTL6B.3	77.5	5	RRT, GKC, YPC, FHB, RRT	76.0	78.9	IACX4889	442,381,268	BS00089580_51	454,883,952
durumMQTL6B.4	90.8	2	RRT	86.9	94.7	wsnp_JG_c1834_901723	537,655,953	Tdurum_contig44825_307	588,937,432
durumMQTL6B.5	104.7	2	LS, NDVI	103.4	105.9	TA004372-0730	621,526,724	BS00011523_51	633,371,119
durumMQTL6B.6	127.2	3	PGC	126.5	128.0	BS00109717_51	662,889,085	Tdurum_contig45914_283	663,681,523
durumMQTL7A.1	61.6	5	LR, SPAD, PDL, NDVI	60.4	62.7	Tdurum_contig31137_373	61,412,931	RAC875_c10701_435	65,969,594
durumMQTL7A.2	102.3	2	FHB, YPC	98.3	106.3	BobWhite_c48548_106	131,332,420	Tdurum_contig51089_1066	163,557,572
durumMQTL7A.3	114.7	2	RRT	109.1	120.3	RFL_Contig5676_748	200,976,557	BS00069163_51	511,685,823
durumMQTL7A.4	145.4	4	YPC, Fb, SR, RRT	142.8	147.9	BS00044234_51	631,404,872	BS00022202_51	641,161,271
durumMQTL7A.5	175.7	3	YPC, PGC	173.7	177.7	wPt-5558	682,897,955	Tdurum_contig31699_276	691,003,050
durumMQTL7A.6	179.6	2	AX, YPC	179.5	179.8	Tdurum_contig31699_276	691,003,050	Tdurum_contig31699_276	691,003,050
durumMQTL7B.1	2.3	5	RRT, GPC, FHB	0.1	4.6	Ex_c21249_1111	886,966	Tdurum_contig49737_462	5,096,321
durumMQTL7B.2	29.7	4	FHB, CIR, GseY	27.6	31.9	RAC875_c10672_440	87,960,765	wsnp_Ex_c36325_44308589	53,938,632
durumMQTL7B.3	52.9	4	RRT, YPC	51.5	54.3	BS00000170_51	103,606,730	Excalibur_c1694_899	105,323,515
durumMQTL7B.4	75.4	2	GseC, Fb	72.7	78.1	Kukri_c9353_642	255,076,815	RAC875_c22594_125	388,539,117
durumMQTL7B.5	89.2	2	GSC, AX	84.8	93.6	CAP8_c949_312	437,505,136	Kukri_rep_c71356_236	512,177,803
durumMQTL7B.6	116.4	3	LR, YPC, YR	113.8	119.0	RAC875_c18043_411	578,959,591	Excalibur_c58742_144	593,689,787
durumMQTL7B.7	137.0	3	GSeC, SR	134.9	139.2	wsnp_Ex_c10307_16890310	630,702,498	Kukri_c31628_571	641,717,556
durumMQTL7B.8	172.8	3	YPC, RRT	170.7	174.8	wsnp_Ex_rep_c101269_86663549	684,341,861	wsnp_Ex_c2365_4431185	687,868,244
durumMQTL7B.9	206.53	7	YPC, LR, Fb, SR, GPC	206.5	206.6	RAC875_rep_c106035_443	715,557,101	Tdurum_contig28601_486	716,329,509

The number of MQTL per chromosome ranged from four in chromosomes 1A and 6A to 9 in chromosome 7B. Chromosomes 3A and 7A showed the highest number of individual QTL (4), chromosome 7B the highest number of undefined QTL (4). The number of QTL per MQTL ranged from 2 in 26 MQTL to 11 in the *durum*MQTL2B.7.As 41 MQTL (47%) derived from the clustering of QTL from threeor more different studies on different parental lines, they will be more stable across environments. The number of traits involved in each MQTL ranged from 1 in twelveMQTL to 7 in the MQTL *durum* MQTL1B.3. Six MQTL involved 5or more different traits (Table 3). The CI of the MQTL ranged from 0.1 to 14 cM, with an average of 4.9 cM. This isa significant reduction from the original QTL, whichranged from 0.4 to 108.1 cM, with an average of 25.5 cM.

The three criteria proposed by Löffler et al.^14^ were used toidentify the most promising MQTL for marker-assisted selection and candidate gene analysis: (1) small MQTL supportintervals, (2) large number of initial QTL and (3) high PVE values of the original QTL. A total of 17 MQTL were selected using the following criteria: a number of QTL per MQTL equal to or greater than 5, with a CI equal toor lower than the average (4.9), and a mean PVE value for the original QTL in the MQTL equal to or greater than 0.10 (Table 4).Only MQTL with a physical distance of less than 5 Mb were subsequently selected for candidate gene (CG) identification.

**Table 4:** Selected MQTL.

MQTL	QTL	CI (cM)	Distance between flanking markers (Mb)	PVE original QTL
durumMQTL2B.1	5	0.8	1.4	0.41
durumMQTL2B.8	5	0.4	0.5	0.10
durumMQTL3A.4	5	2.6	1.4	0.11
durumMQTL3B.1	7	1.7	0.7	0.11
durumMQTL3B.5	5	4.4	4.6	0.13
durumMQTL6A.3	6	3.5	1.3	0.10
durumMQTL6A.4	6	0.4	0.3	0.10
durumMQTL6B.1	5	4.5	4.1	0.10
durumMQTL7A.1	5	2.3	4.6	0.22
durumMQTL7B.9	7	0.1	0.8	0.19

### Candidate genes and in silico gene expression analysis

Candidate genes (CG) for investigating and estimating relative gene expression levels were identified within the MQTL regions reported in Table 4. The flanking markers of the CI were launched against the genome browser for both ‘Svevo’ (durum wheat)^34^ and ‘Chinese spring’ (bread wheat) (https://iwgs.org/) reference genomes. Excluding transposable elements, atotal of 436 and 326 gene modelswere detected for ‘Svevo’ and ‘Chinese Spring’ respectively (Additional file 1). Differentially expressed genes (DEG) upregulated under abiotic and biotic stress conditions (Table 5) and expressed in the grain tissues for quality CGs were subsequently analysed using the RNAseq data available at http://www.wheat-expression.com/^35^.

**Table 5:** Number of genes detected for each MQTL.

MQTL	Number of genes	
	DURUM wheat	Bread wheat
durumMQTL2B.1	29	42
durumMQTL2B.8	8	11
durumMQTL3A.4	24	32
durumMQTL3B.1	20	22
durumMQTL3B.5	111	45
durumMQTL6A.3	20	8
durumMQTL6A.4	16	4
durumMQTL6B.1	107	69
durumMQTL7A.1	104	69
durumMQTL7B.9	17	24

**Figure 4:**
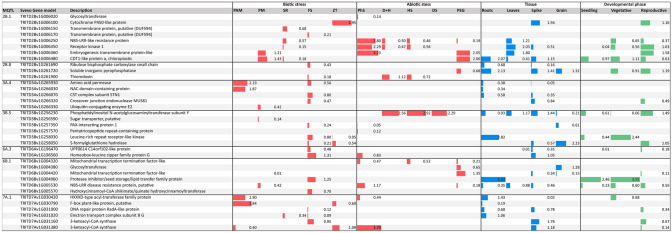
Expressed genes involved in biotic and abiotic stress, in different tissues and developmental phases for each MQTL.

Thebread wheat gene models were analysed using the RNAseq experiments available at www.wheat-expression.com^35,36^. In particular, the study focused on identifying expression genes involved in biotic and abiotic stress, in different tissues and developmental phases (Fig. 4).

A total of 36 CGs upregulated under biotic and abiotic stress were found in seven MQTL. MQTL3B.1 and MQTL7B.9 in ‘Svevo’ and ‘Chinese spring’ did not yieldhomologous gene models, and no upregulated gene models were found for MQTL6A.4 (Fig. 4).

The genes most expressed during biotic stress conditions with respect to control conditionswithout stress were: (1) for expression analyses using pathogens associated molecular patterns (PAMP), F-box plant-like protein (7A.1), amino acid permease (3A.4), HXXXD-type acyl-transferase family protein (7A.1) and NAC domain-containing protein (3A.4); (2) for powdery mildew infection, CDT1-like protein and embryogenesis transmembrane protein (2A.1);( 3) for infection with *Fusarium pseudograminearum*, homeobox-leucine zipper family protein G (6A.3), protease inhibitor/seed storage/lipid transfer family protein (6B.1) and 3-ketoacyl-CoA synthase (7A.1); and (4) for infection with *Zymoseptoriatritici*, cytochrome P450-like protein (2B.1) and 3-ketoacyl-CoA synthase (7A.1) during stress.

Expression analysis under abiotic stress included: phosphorous starvation, drought stress, heat stress, combined drought and heat, addition of PEG 6000 to simulate drought and cold stress. No upregulated genes were found for cold stress. The most expressed genes identified associated to phosphorus starvation conditions were NBS-LRR disease resistance proteins (2B.1 and 7A.1), receptor kinase 1 (2B.1), embryogenesis transmembrane protein-like (2B.1) and 3-ketoacyl-CoA synthase (6B.1).

Phosphatidylinositol N-acetylglucosaminyltransferase subunit Y (3B.5) was differentially expressed under combined drought and heat and under those single conditions, whereas a thioredoxin (2B.8) was also expressed under combined drought and heat stress. Lastly, three genes were the most upregulated when simulating drought stress using PEG:CDT1-like protein a, chloroplastic (2B.1), embryogenesis transmembrane protein-like (2B.1) and mitochondrial transcription termination factor (6B.1).

According to the plant tissue where the genes were upregulated, the spikes showed the higher number of transcripts with expression levels higher than 1tpm (7), whereas the lower number was found in grain (3). From below ground to the top of the plant, the most expressed gene models were: (1) roots: protease inhibitor/seed storage/lipid transfer family protein (6B.1), leucine-rich repeat receptor kinase (3B.5), soluble inorganic pyrophosphatase (2B.8), CDT1-like protein (2B.1), HXXXD-type acyl-transferase family protein (7A.1), electron transport complex subunit B G (7A.1) and phosphatidylinositol N-acetylglucosaminyltransferase subunit Y (3B.5); (2) leaves: receptor kinase 1, Embryogenesis transmembrane protein-like, NBS-LRR-like resistance protein (2B.1), phosphatidylinositol N-acetylglucosaminyltransferase subunit Y (3B.5) and NBS-LRR disease resistance protein (6B.1); 3) cytochrome P450-like protein (2B.1), 3-ketoacyl-CoA synthase (7A.1), phosphatidylinositol N-acetylglucosaminyltransferase subunit Y (3B.5), soluble inorganic pyrophosphatase (2B.8), 3-ketoacyl-CoA synthase (7A.1), CDT1-like protein (2B.1), homeobox-leucine zipper family protein G (6A.3) and crossover junction endonuclease MUS81 (3A.4); and (4) grain: S-formylglutathione hydrolase (3B.5), soluble inorganic pyrophosphatase (2B.8) and glycosyl transferase (6B.1).

Three developmental phases were considered in expression analysis: seedling, vegetative, and reproductive. The most expressed genes in seedlings were protease inhibitor/seed storage/lipid transfer family protein (6B.1) and CDT1-like protein (2B.1), whereas during vegetative growth they were protease inhibitor/seed storage/lipid transfer family protein (6B.1), leucine-rich repeat receptor-like kinase (3B.5), CDT1-like protein (2B.1) and soluble inorganic pyrophosphatase (2B.8). Lastly, during the reproductive stage the most expressed gene models were receptor kinase 1, embryogenesis transmembrane protein (2B.1), phosphatidylinositol N-acetylglucosaminyl transferase subunit Y (3B.5), soluble inorganic pyrophosphatase (2B.8), cytochrome P450 protein (2B.1) and S-formylglutathione hydrolase (3B.5).

Gene expression in grains was analysed not only under biotic or abiotic stress conditions but also to detect candidate genes of importance in grain quality.

High expression levels (tpm > 2) were observed in grain for phospholipid-transporting ATPase, nascent polypeptide-associated complex subunit alpha-like protein, acetyltransferase component of pyruvate dehydrogenase complex, polyadenylate-binding protein-interacting protein 4, acyl-CoA N-acyltransferase with RING 2FFYVE 2FPHD-type zinc finger protein, mitochondrial inner membrane protease subunit 1, S-formylglutathione hydrolase and peroxidase (on 3B.5, 6A.3 and 6B.1).

When grain tissues ofthe endosperm, embryo, aleurone layer, seed coat and transfer cells were dissected, all the genes described above for the whole grain were strongly expressed in at least one of the different tissues. Other gene models that expressed over 2 tpm were: glycerol-3-phosphate dehydrogenase [NAD( +)] in the aleurone layer and seed coat, a 28S ribosomal S34 protein in the embryo, S-acyltransferase in the aleurone layer, a pimeloyl-[acyl-carrier protein] methyl ester esterase in the aleurone layer, glycosyltransferase in the endosperm, hydroxyproline-rich glycoprotein-like G in the aleurone layer and seed coat, histidine-containing phosphotransfer protein in the embryo, a general regulatory factor 1G in the embryo, aleurone layer and seed coat, S-adenosyl-L-methionine-dependent methyltransferase superfamily protein in the seed coat, an F-box in the aleurone layer, and phosphatidylinositol N-acetylglucosaminyl transferase subunit Y in the endosperm, embryo and seed coat.

## Discussion

One of the main challenges of breeding programs is to increase crop yield. Crop productivity is highly affected by environmental constraints and diseases, so thatnew cultivars must incorporate new loci to cope with the different stresses affecting plant growth and yield. Breeders have another important challenge in the development of new cultivars: to improve grain quality for end products that meet industrial and consumer requirements.

In recent years numerous studies have been carried out to identify new loci controlling traits for abiotic and biotic stress tolerance and grain quality in bread and durum wheat. QTL meta-analysis has been carried out on most of the QTL identified in durum wheat for disease resistance, environmental tolerance and grain quality. This approach has been used extensively in plants since its development in 2004^37^. It is especially useful in detecting major loci for quantitative traits and, by increasing map resolution, in identifying candidate genes controlling polygenic traits^12^.

This is the first study that provides an overview and comparison of genetic loci controlling multiple traits in durum wheat, including quality traits and biotic and abiotic traits. It adds new MQTL for durum grain traits: some of the MQTL were mapped with high precision and are relatively more robust and stable with major effects.

We report a total of 368 QTL distributed on all 14 chromosomes, of which 171 are related to quality traits, 127 to abiotic stress, and 71 to biotic stress, over a total of 34 mapping population. A total of 85 meta-QTL were identified, of which 15 meta-QTL were selected as the most promising for candidate gene selection.

The meta-analysis conducted in this study accurately compared genomic positions of individual QTL identified in different studies and refined the confidence intervals of the main genomic regions associated with different traits. The durum wheat consensus map^15^ preserved the marker order of individual maps, and confidence intervals were calculated to highlight differences between the original map position and its projection. For abiotic stress and quality traits, there was a reduction in the CI, whereas biotic stress traits showed an increase in the confidence interval. This may be due to the quantitative nature of the different traits; individual QTL for abiotic stress and quality showed lower PVE values, whereas those related to disease resistance yielded higher values (means of 0.11, 0.12 and 0.20 respectively). Biotic stress traits were controlled by a lower number of genes than traits related to abiotic stress or quality. Results reveal that the number of QTL per study was 25 for abiotic stress traits, 12 for quality related traits and 3 for biotic stress traits. Comparison of the reduction of CIs and number of genome regions involved in trait variation between this study and other studies carried out in durum wheat (quality)^30^, bread wheat (abiotic and biotic traits)^13,29^ and maize (yield)^38^ is reported in Additional file 3. Reduction of the CI and number of QTL after meta-analysis was 80% and 77% respectively, which is within the range among the different studies (from 60 to 88% for CI and from 65 to 90% for number of QTL).

The MQTL identified provide more closely linked markers due to the availability of a durum wheat consensus map^15^. Some of these are also linked to known major genes for other agronomically important traits, there by adding value to these MQTLas targets for marker assisted selection using the SNP markers flanking the MQTL, however an initial validation of the alleles reporting favourable effects should be addressed. According to the genome position of important agronomic genes reported in Liu et al.^39^, eleven MQTL were found to include 12 genes enhancing grain yield, quality, or plant development. DurumMQTL5A.5 and durumMQTL7B.9 included the vernalization genes *Vrn*-A1 and *Vrn*-B3 respectively. The incorporation of favourable alleles for this gene during breeding helps develop spring habit without cold requirements for flowering^40^, thus can be used as a strategy for introgressing important target traits from non-adapted pre-breeding materials combining the most favourable vernalization alleles. DurumMQTL4B.4 carries the dwarfing gene *Rht*-B1. Dwarfing genes were the basis of the green revolution, allowing an up to 35% increase in the yield of durum wheat^41^. Five durumMQTL, 2B.7, 4A.1, 7A.1, 7A.2 and 7A3, included genes involved in grain weight and size, the genes *TaGS2*-B1, *TaCwi*-A1, *TaTEF*-7A, *TaGASR7*-A1 and *TaTGW*-7A. Other genes affecting grain yield and quality were the *TaSdr-*A1 and *TaALP*-4A involved in preharvest sprouting tolerance and located in durumMQTL2A.4 and durumMQTL4A.5, respectively. Preharvest sprouting is an important limiting factor for grain yield in the major wheat production areas, especially when frequent rainfall occurs during harvest. Lastly, two genes involved in grain quality were found in durumMQTL1A.1 (*Glu*-A3) and durumMQTL7B.9 (*Psy*-B1). According to Subirà et al.^42^, the introgression of favorable alleles for HMW and LMW glutenin subunits led tothe improvement of pasta-making quality in modern durum wheat cultivars. The phytoene synthase gene *Psy*-B1 is involved in the biosynthesis of carotenoid pigments.

An interesting case of study was in the durumMQTL2B.1 where are co-located QTL for RRT (abiotic stress) and SBCMV (biotic stress). Looking at candidate gene reported in Fig. 4, NBS-LRR-like resistance genes were highly expressed in both abiotic and biotic stresses experiments, which may indicate a link between the two traits and a pleiotropic effect on root development and pathogen growth. This theory has been supported by Kochetov et al.^43^, which reported a differential expression of NBS-LRR-encoding genes detected in the root transcriptomes of two *Solanumphureja*.

The most promising MQTL arethe ones located on chromosome 1B (two MQTL), 2B (three MQTL), 3A (1 MQTL), 3B (two MQTL), 5B (1 MQTL), 6A (two MQTL), 6B (two MQTL), 7A (1 MQTL) and 7B (1 MQTL). These showed co-localized QTL for several grain traits, as found in earlier studies on bread wheat^44–46^, indicating that QTL are not randomly spread throughout the genome but cluster in specific genomic regions. The study of different MQTL has revealed how some traits are always associated, such as FHB, GPC and YPC (durumMQTL1B.3, durumMQTL1B.5 and durumMQTL6B.3) or RRT, SPAD and NVDI (durumMQTL2B.1, durumMQTL3B.1, durumMQTL6A.4, durumMQTL7A.1). This represents an important key for identifying and characterizing genes associated with the MQTL, with a pleiotropic effect on yield-related traits and quality traits.

To correlate between MQTL and previous QTL identified by GWAS, MQTL positions were compared with marker trait associations (MTA) reviewed by Colasuonno et al.^11^ for abiotic and biotic stress and quality traits. Of the 352 MTA, 58 were located within 33 durum MQTL. Of these, 37 MTA in 26 MQTL reported associations with one of the traits included in the MQTL (Additional file 2). The highest number of MTA per trait category corresponded to LR for biotic stress, NDVI for abiotic stress and YPC for grain quality. These MTA were distributed in 11 chromosomes. These results suggest that new bioinformatic tools are required to integrate association studies with QTL meta-analysis for better understanding the molecular bases of trait variation in crop species.

## Conclusions

QTL meta-analysis can help validate QTL previously detected in different populations and unravel the most stable QTL for the most important wheat traits. This studyused QTL meta-analysis toacquirea comprehensive picture of the mainregions of the durum wheat genome involved in the control of multiple traits so as to identify QTL-enriched regions and candidate genes with possible pleiotropic effects.

The numerous markers within stable QTL and rich candidate gene regionscan helpelucidate the mechanism regulatingmany traits and speed up breeding programs for the production of top-quality cultivars.

## Material and methods

### Collection of QTL database and projection on a consensus map

A thorough bibliographic review was carried out on the literature reported in Colasuonno et al.^11^. QTL information on biparental durum wheat populations was retrieved from 41 independent studies, including a total of 36 different traits (Table 1) relating to quality (14), biotic stress (22) and abiotic stress (5).

Information on chromosome location, the most closely flanking markers, QTL position, logarithm of odds (LOD) values, confidence intervals (CIs) and phenotypic variance explained (PVE or r^2^) values are summarized in the review by Colasuonno et al.^11^.

To representall the QTL in one linkage map, the durum wheat consensus map developed by Maccaferri et al.^15^ was used for QTL projection, following the homothetic approach described by Chardon et al.^37^ as described in Colasuonno et al.^11^. The CIs for the projected QTL were estimated for a confidence interval of 95% using the empirical formula proposed by Guo et al.^47^.

### QTL meta-analysis

QTL meta-analysis was conducted using BioMercator v.4.2^48^, available at https://urgi.versailles.inra.fr/Tools/BioMercator-V4, adopting the approach developed by Veyrieras et al.^49^. Meta-analysis determines the best QTL model based on model choice criteria from the Akaike information criterion (AIC), a corrected AIC, a Bayesian information criterion (BIC) and the average weight of evidence (AWE). The best QTL model was selected when the lowest values of the model selection criteria were achieved in at least threemodels. Consensus QTL from the optimum model were regarded as MQTL.

### Identification of candidate genes underlying the MQTL region and expression analysis

Gene models within MQTL were identified using the high-confidence genes reported for the durum wheat reference sequence^34^, available at https://wheat.pw.usda.gov/GG3/jbrowse_Durum_Svevo based on the positions of markers flanking the CI of the MQTL.

In silico expression analysis and the identification of upregulated gene models was carried out using the RNAseq data available at http://www.wheat-expression.com/^35^ using gene models, from ‘Chinese spring’, located within the markers flanking the MQTL (https://iwgs.org/). Homologous genes from ‘Svevo’ were subsequently identified in durum wheat.

## Supplementary Information

Supplementary Information 1.

Supplementary Information 2.

Supplementary Information 3.

## Data Availability

All data generated or analysed during this study are included in this published article [and its supplementary information files].
