# Supplementary material for: Meta-QTL analysis and identification of candidate genes for quality, abiotic and biotic stress in durum wheat
Source: Sci Rep. 2021 Jun 4;11:11877. doi: 10.1038/s41598-021-91446-2 (PMC8178383; doi:10.1038/s41598-021-91446-2)
Supplement: Supplementary file 3 — Supplementary Information 3. [file 41598_2021_91446_MOESM3_ESM.docx]

| **Trait** | **Species** | **Reference** | **CI original** | **N projected QTL** | **CI MQTL** | **N MQTL** | **CI reduction** | **N reduction** |
| --- | --- | --- | --- | --- | --- | --- | --- | --- |
| Several | Durum wheat | Current study | 25.5 | 368 | 4.9 | 85 | 80% | 77% |
| Leaf rust | Bread wheat | [29] | 20.8 | 144 | 8.4 | 35 | 60% | 76% |
| Roots | Bread wheat | [13] | 14.8 | 634 | 4.9 | 94 | 67% | 85% |
| Quality | Durum wheat | [30] | 18.0 | 249 | 6.4 | 45 | 82% | 65% |
| Yield | Maize | [38] | 26.4 | 808 | 3.1 | 84 | 88% | 90% |
| **Mean** | **-** | **-** | **21.1** | **441** | **5.5** | **69** | **75%** | **79%** |
